# Nurse Motivation, Engagement and Well-Being before an Electronic Medical Record System Implementation: A Mixed Methods Study

**DOI:** 10.3390/ijerph18052726

**Published:** 2021-03-08

**Authors:** Rebecca M. Jedwab, Alison M. Hutchinson, Elizabeth Manias, Rafael A. Calvo, Naomi Dobroff, Nicholas Glozier, Bernice Redley

**Affiliations:** 1 Monash Medical Centre Clayton, Monash Health Digital Health Division, Nursing and Midwifery Informatics, Melbourne, VIC 3168, Australia; naomi.dobroff@monashhealth.org; 2 Faculty of Health, School of Nursing and Midwifery, Melbourne Burwood Campus, Deakin University, Melbourne, VIC 3125, Australia; emanias@deakin.edu.au; 3 Centre for Quality and Patient Safety Research—Monash Health Partnership, Deakin University, Melbourne, VIC 3168, Australia; alison.hutchinson@deakin.edu.au (A.M.H.); bernice.redley@deakin.edu.au (B.R.); 4 Dyson School of Design Engineering, Imperial College London, South Kensington, London SW7 2DB, UK; r.calvo@imperial.ac.uk; 5 Central Clinical School, Faculty of Medicine and Health, Sydney School of Medicine, The University of Sydney, Sydney, NSW 2050, Australia; nick.glozier@sydney.edu.au

**Keywords:** nursing, nursing workforce, electronic medical record, motivation, work engagement, well-being, burnout, health communication

## Abstract

Implementation of an electronic medical record (EMR) is a significant workplace event for nurses in hospitals. Understanding nurses’ key concerns can inform EMR implementation and ongoing optimisation strategies to increase the likelihood of nurses remaining in the nursing workforce. This concurrent mixed-methods study included surveys from 540 nurses (response rate 15.5%), and interviews with 63 nurses to examine their perceptions of using a new EMR prior to implementation at a single healthcare organisation. Survey findings revealed 32.2% (*n* = 174) of nurses reported low well-being scores and 28.7% (*n* = 155) were experiencing burnout symptoms. In contrast, 40.3% (*n* = 216) of nurses reported high work satisfaction, 62.3% (*n* = 334) had high intentions of staying in their role, and 34.3% (*n* = 185) were engaged in their work. Nearly half (*n* = 250, 46.3%) reported intrinsic motivation towards EMR use. Thematic analysis of focus group interviews revealed two themes, each with three subthemes: (1) *Us and Them*, detailed the juxtaposition between nurses’ professional role and anticipated changes imposed on them and their work with the EMR implementation; and (2) *Stuck in the middle*, revealed nurses’ expectations and anticipations about how the EMR may affect the quality of nurse-patient relationships. In conclusion, anticipation of the EMR implementation emerged as a stressor for nursing staff, with some groups of nurses particularly vulnerable to negative consequences to their well-being.

## 1. Introduction

Implementation of a new healthcare technology, such as an electronic medical record (EMR) system is a significant investment for healthcare organisations and systems, that may pose risks for the quality of healthcare delivery and workers themselves. The engagement, motivation and well-being of nurses is critical for successful EMR implementation, and a high-performing nursing workforce to deliver safe, high quality patient care.

EMR systems are replacing paper-based systems worldwide for recording patient care [1]. Recently, EMR systems are a key facilitator for clinical documentation, clinical decision-making and information management in healthcare organisations. EMRs do more than digitise pre-existing paper forms; they alter nursing work and workflows for when, where and how care delivery is documented [2,3]. EMR systems are expected to improve nurses’ work by making it more streamlined and efficient, however technology-related changes to nurses’ work and workflows have a wide range of impacts [4,5]. Changes to nurses’ work and workflows, inter- disciplinary and intra-disciplinary communication, and nurses’ well-being may have negative impacts on quality-of-care delivery and patient safety outcomes [2,3,6,7]. Factors that influence nurses’ adoption, and therefore EMR effectiveness, include system factors (e.g., type and scope of the system), organisational leadership and local work culture [6]. However, the role of individual nurse factors such as motivation, engagement, well-being, burnout, and job satisfaction are not often considered.

Motivation to change a person’s behaviour, such as use of a new technology at work, is affected by intrinsic and extrinsic factors, as well as their autonomy and self-efficacy [8]. Examining nurses’ autonomy in relation to EMR adoption assists with understanding their motivation to engage in, accept and adopt behaviours [9], and understanding nurses’ self-efficacy and self-regulation for EMR implementation can reveal important factors for nurses’ motivation to use the EMR [10].

Motivation and workplace well-being are strong influences on work engagement [11]. Nurses’ work engagement is reflected in positive career involvement or behaviours, while reduced nurse work engagement has negative impacts on patient safety, decreases job satisfaction and increases work turnover [12,13,14]. Literature has often focused on examining nurses’ adoption of specific EMR tools, processes or workflows; work engagement is seldom examined [15]. Nurses’ general attitudes, perceptions and opinions towards EMRs have been mostly positive [16] but factors influencing their engagement in EMR adoption remain underexplored.

Workplace well-being is a subjective measure of an individual’s positive health, emotions and satisfaction [17]. Among nursing and medical professionals, workplace well-being has positive effects on work engagement and performance, and an important role in patient safety and quality outcomes [17,18]. Conversely, poor well-being is linked to worker burnout [19,20]. Nurses’ and doctors’ well-being in conjunction with EMR implementation have often been examined using satisfaction as a proxy, in the context of specific clinical processes or workflows, such as clinical handover [21], overall EMR usability [22,23], electronic prescribing [24], or from patients’ perspectives [25].

Burnout is a measurable syndrome that develops from prolonged exposure to physical or psychological stressors [26], and is well documented among healthcare professionals. Burnout decreases motivation and work engagement, and contributes to both physical and psychological health complications [27]. Psychosocial stressors for nurses include complex healthcare environments, and clinical and patient safety concerns [28,29,30]. Specific occupational groups of nurses (e.g., emergency department and critical care) are particularly vulnerable to burnout [31,32]. Burnout among nurses is costly to healthcare organisations [33] due to negative consequences for the quality of patient care, as well as individual nurse and work outcomes [34]. Both well-being and burnout are multi-factorial, and directly impact the individual, their work satisfaction, intention to stay and work engagement [35,36]. Nurse burnout is associated with low resilience, low job satisfaction and reduced well-being [28,29,30]. Cynicism, a component of burnout, is an important mediating factor in nurses leaving their jobs [29]. Current and projected nurse workforce shortages make it important to examine, and mitigate where possible, factors that affect nurses’ intentions to remain, or leave the workforce [37,38].

Few studies examining the impact of EMRs have included the influences on nurses themselves [39]. Emerging evidence suggests EMR-related factors were a cause of stress and frustration for nurses, which may be associated with higher burnout [40]. Findings of another study reported nurse turnover significantly increased, and did not return to baseline, one year post-EMR implementation [41]. Whilst studies examining EMR impacts on nurses are limited, the impacts of EMRs on physicians’ well-being are well-documented. Researchers have attributed EMR-related burnout among physicians to the negative impacts of stress and prolonged EMR use [42,43,44,45]. Nurses and physicians raised similar concerns about EMR usability [46], however the potential negative impacts of an EMR implementation on the nursing workforce have not been systematically explored. As the largest users of EMRs in hospitals, nurses are expected to be similarly, if not more, susceptible to EMR-related burnout and negative consequences than physicians. The potential detrimental effects of burnout such as low engagement, low well-being and high turnover, makes examining the impact of EMR implementation on nurses an important concern.

Work satisfaction is a complex phenomenon that varies in different settings according to individual values [47] and is often linked to burnout, nurse turnover and missed nursing care [48]. Nurses’ work satisfaction mediates their intention to leave the workforce [49], and contributes to negative emotional, physical, psychological and financial consequences for individual nurses, patients and organisations [50]. One previous study examined nurses’ work satisfaction with an EMR system, but this only related to satisfaction with specific clinical components of the system such as documentation or medication administration [51]. A gap exists in current understanding of the complex factors associated with nurses’ work satisfaction with EMR systems.

Qualitative examinations of nurses’ satisfaction, engagement and retention provide a comprehensive understanding of the many challenges and changes that an EMR implementation creates for nurses [52]. Majority of the studies examining nurse perceptions and adoption of EMR systems relate to specific features, or functions of nursing work [53,54], rather than the broader implementation process. Deep understanding of nurses’ perceptions during implementation of a new EMR system can assist development of deployment and optimisation processes to enhance nurse motivation, engagement and well-being, which in turn, enhances nurse and organisational performance, and safety and quality of patient care.

### 1.1. Theoretical Framework

Multiple theories have been developed to explain the numerous components of human behaviours or actions related to the use or acceptance of technology. For example, the technology acceptance model (TAM) proposes perceived usefulness (PU) and perceived ease of use (PEU) affect an individual’s adoption of a particular technology [55]. Use of PU and PEU to examine nurses’ acceptance of an EMR system was unsuitable for this study due to the TAM model’s limitation of assuming that human behaviour is rational and planned, and its failure to incorporate social and cultural aspects that influence an individual’s decision-making [56,57,58]. More recently, the Motivation, Engagement and Thriving in User Experience (METUX) model has been developed to incorporate understanding of complex human behaviours. Grounded in psychological research, METUX proposes the use of technology is influenced by an individual’s autonomy, competence and relatedness to the task [10]. This model attempts to provide insights into how an individual’s perceptions of technology can support or undermine basic psychological needs in the workplace, thereby impacting their motivation and engagement, and ultimately, user well-being. This model addresses key concepts explored in this study, that were derived from literature relevant to broad range of potential influences on nurses’ adoption of technology. The novel mixing of qualitative and quantitative data used in this study provided a broad context to examine the key concepts, hence was expected to provide comprehensive understanding of how nurse motivation, engagement and well-being are associated with an EMR implementation.

### 1.2. Significance

The success of an EMR implementation must consider not only the suitability, feasibility and acceptability of the system to nurses as the largest group of end users, but also the potential for impacts of the system on nurses themselves [59,60]. Literature suggests potential for both positive and negative consequences of an EMR implementation for nurses’ motivation, engagement and well-being at work; however, these three concepts have not been investigated in relation to this phenomenon, either concurrently with implementation or over time. Understanding nurse motivation, engagement and well-being may be useful to predict not only the success of EMR adoption by nurses into the workplace, but also to identify potential risk for unintended physical, psychological, and emotional stress in nurses. Research is needed on the consequences (intended or otherwise) of EMR implementations for nurses [61]. This gap in the literature hinders understanding of the extent to which EMR systems influence nursing work, and nurses themselves. Understanding the breadth and depth of these impacts is paramount to promoting a strong workforce, while also minimising potential for negative impacts on nurse attrition and work performance due to EMR implementation.

### 1.3. Purpose

The purpose of this study was to identify factors important for successful EMR adoption by nurses, and identify vulnerabilities requiring specific attention to support implementation and adoption success. The outcomes of this work are expected to inform evidence-based strategies to support nurses with the EMR implementation, as well as strategies for subsequent sustained use and optimisation.

## 2. Materials and Methods

### 2.1. Design

The natural experiment of an organisation-wide EMR implementation in 2019 was used for this study. Using a concurrent mixed-methods design, complementary qualitative (focus group interviews) and quantitative (survey) data were given equal importance to comprehensively examine this complex topic [62]. The two data sources provided methods triangulation, whereby complementary data were collected and analysed to enrich the research conclusions [63].

### 2.2. Setting

The study setting was a large, multi-site tertiary teaching hospital located in Victoria, Australia with approximately 3300 beds over seven major hospitals. The health service provided care across a large geographic area through a mix of in-patient, ambulatory, residential and community health settings. Patient populations included specialist, critical care, acute, sub-acute and community of all ages from neonates, paediatric, adult and the elderly. As of January 2019, the healthcare organisation employed almost 8400 nurses and midwives and had well-established relationships with multiple universities for teaching and research.

### 2.3. Participants and Sample

A census sampling approach was used to recruit all eligible nursing staff, with varying demographics, from different hospital sites within the healthcare organisation and who work in a range of clinical areas that provide care to acute, sub-acute, adult, paediatric and neonatal patients, including specialty inpatient settings. It was expected that sampling a broad range of nursing staff would provide a representative sample of the wider nursing population of this healthcare organisation, and increase the transferability of the study findings. Six of the seven hospital sites where initial EMR implementation occurred sequentially throughout 2019 were selected. Hospital administrative records identified 3479 nurses working in the eligible sites at the commencement of data collection. Data collection occurred between January and November 2019. Data collection ended when EMR use commenced. A desired response rate of 25–30% was expected to provide a representative sample [64].

#### 2.3.1. Inclusion Criteria

All nurses working in inpatient units at the six sites where the EMR system was implemented were eligible for inclusion.

#### 2.3.2. Exclusion Criteria

Areas included in the initial EMR implementation as hybrid workflows, where a mix of EMR and paper documentation would be used, were excluded as the extent to which nurses in these areas would be using the EMR in their work and workflows could not be determined. In this study this meant maternity, mental health and ambulatory care units were excluded. Casual employees were also excluded as it was not possible to determine if these staff were actively working within the organisation, the areas in which they were working, their participation in the EMR training, or their recency of practice. In addition, members of the EMR implementation team were excluded to avoid bias related to their roles in development, training and establishment of the EMR system.

#### 2.3.3. Recruitment

The survey was accessible via an email link sent to individual nurses by nurse managers, a quick response (QR) code on study advertising information materials in wards (which allowed direct mobile phone access to the online survey), or paper surveys delivered to wards in-person (R.J.) at least twice. The survey preamble provided study information and consent was indicated by submitting a completed survey. Strategies recommended by Dillman et al. were used to enhance survey responses such as including both paper and online response options, email communication with nursing staff containing links to the survey, providing QR codes in staff accessible areas, and keeping the survey questions as short as possible [64].

Recruitment of a convenience sample for qualitative focus group interviews involved inviting nurses to indicate their interest by including their email contact at the end of the survey, and a subsequent invitation from the research team. In addition, in-person recruitment occurred during visits to the clinical areas at each site. Written consent was obtained prior to focus group data collection.

### 2.4. Survey

Survey data were collected and managed using Research Electronic Data Capture (REDCap) software version 6.14.1 (REDCap, Nashville, USA), hosted securely by the University [65]. The survey contained seven validated tools to measure the study concepts. Wording of items in the ACTA tool was adapted by replacing ”technology” with ”electronic medical record”. Table 1 details the study concepts and survey measurement tools, the number of items and scoring for each.

#### 2.4.1. Quantitative Data Analysis

Data were extracted securely, and analysis was undertaken using IBM SPSS^®^ Statistics for Windows software, Version 24 (IBM Corporation, New York, NY, USA). Data cleaning and examination of missing data were followed by creating scale scores using authors’ instructions, and examination of frequencies and distributions. Descriptive statistics were used for initial reporting and data familiarisation. Tool reliability within the study population was examined using Cronbach’s alpha. Relationships between variables were examined using bivariate correlations, and Chi-square analyses were used to examine relationships between groups. Significance was set at *p* < 0.05, and the bootstrapping method (sampling repeated 1000 times) was adopted to construct 95% confidence intervals (CIs) for significance testing of the indirect effects (considered significant when the 95% CI did not include zero) [72].

#### 2.4.2. Piloting the Survey

Prior to distribution, the survey was peer-reviewed by experts in the fields of nursing, psychology and informatics with minor formatting and item order changes made. Next, 12 nurses expected to represent the target population, but not eligible to participate in the study due to their roles in the research or the EMR team, then pilot tested the survey. These nurses examined the feasibility of the survey completion modes (online and paper), evaluated survey length and flow (logic of order of items and duration to complete), clarity of the wording and suitability of the data for the proposed analysis [64]. No changes were required after piloting testing.

### 2.5. Focus Group Interviews

Qualitative data were collected to provide in-depth understanding of nurses’ perceptions of the EMR implementation, and contextual insights into the complex interactions between motivation, engagement and well-being before the EMR implementation [73]. In accordance with ethical approval, an AUD$3.50 drink voucher was offered to each focus group interview participant as a token of gratitude for their participation.

The focus group interviews were held at mutually convenient times and locations to support attendance and were audio-recorded and transcribed for analysis. In all, 63 nurses participated in one of 12 focus group interviews, with 2–9 participants in each group (median = 4.5) which were between 12 and 32 min in duration (median = 23.5). Field notes were collected by an independent observer, and additional reflective notes were made by the interviewer and observer at the end of each focus group interview. These notes included the arrangement of the participants in the room, contributing factors such as interruptions, researcher impressions and reflexive notes. These were also transcribed and used for analyses. 

The semi-structured focus group interview guide used open-ended questions drawn from the ”4 I” model developed for appreciative inquiry into organisational change [74], and designed to provide direction and focus discussion on the EMR implementation, but not restrict discussion to specific issues. Example questions included “What do you think about the EMR?” and “How are you feeling about the EMR implementation?”. Demographic information was collected from all focus group participants using an anonymous paper form with the same items as the survey (detailed in Table 1).

#### Qualitative Data Analysis

Inductive analysis of qualitative data used reflexive thematic analysis [75] to elicit participants’ perceptions and perspectives of EMR implementation. Reflexive thematic analysis was selected as this approach acknowledges the researchers’ active engagement with, and interpretation of, the data [76].

Once transcribed, data were analysed using Braun and Clarke’s six steps of thematic analysis [75]. Listening to the audio recordings and reading the transcriptions and field notes multiple times allowed familiarisation with the data. Two rounds of coding were undertaken inductively in discussion with members of the research team. Codes were grouped into related categories, and these categories were subsequently grouped into subthemes and themes by refining codes at the coded data level, and then at level of the entire data using mind-mapping and consensus by multiple researchers. Themes were named and refined during the discussions with multiple researchers.

### 2.6. Ethics Approval

This study was approved by the healthcare organisation Human Research Ethics Committee (HREC) as low-risk (Reference: HREC/46439/MonH-2018-154603(v3)) and subsequently noted by the university HREC (Reference: 2019-003).

### 2.7. Rigour

The study design incorporates four commonly identified justifications for mixed methods research: (1) triangulation to enable convergence of and corroboration from data sources; (2) complementary elaboration and clarification of research results; (3) exploration of new perspectives; and (4) Incorporation of breadth and range to the data [63]. 

Qualitative research rigor considered credibility, dependability, transferability, and confirmability to establish trustworthiness. Credibility was achieved by member checking and triangulation of the data. Member checking used qualifying questions or restating to check understanding during the focus groups as it was not feasible to contact participants after data collection due to logistics of shiftwork, time burden on participants and their strong desire to remain anonymous. Methods triangulation involved collecting qualitative and quantitative data, and analyst triangulation involved multiple research team members in data analysis to enhance rigor. Broad inclusion criteria, a diverse participant sample and thick descriptions were used to enhance transferability. Confirmability involved the research team, not just an individual researcher, in interpretation of the data. 

Validity of the research was enhanced using data triangulation, descriptive reporting using illustrative examples from the data, ensuring any biases of the research team were explicit, independent checking of transcripts and audio recordings, independent cross-checking and group discussion of codes and themes, and keeping detailed field notes of all decisions. Reflexivity was managed by researchers reflecting on their own perceptions, any biases that may be present and being conscious of the researchers’ roles and relationships to the data [73,77].

## 3. Results

### 3.1. Participant Demographics

Demographic characteristics of the survey and focus group participants were similar; however, among the survey participants there were a higher number of nurses in more senior roles and nurses who had a higher number of years worked. Unfortunately, there were no focus group participants from one site and some nurses who participated in a focus group did not want to provide their demographic information, resulting in some missing data. The demographic characteristics of survey and focus group participants are provided in Supplementary Materials Table S1.

### 3.2. Survey

Of the 556 completed surveys (response rate 15.98%), 58.45% (*n* = 325) were completed online and the remainder used paper (*n* = 231, 41.55%): six met exclusion criteria (nurses working in excluded areas) and a further 10 were excluded from analysis to reduce potential for inaccurate statistical inferences (more than 10% of response fields were incomplete) [70]. A total of 540 surveys were used for statistical analyses.

#### 3.2.1. Survey Tools Assessment

Survey data were examined for normality, scale reliability and score mean and standard deviation (SD).

Testing of Normality (using Kolmogorov-Smirnova) was significant for all survey tools and their dimensions (Work Satisfaction, Intention to Stay, WHO-5, ACTA, UWES-3, MBI and Psychological Safety questions), indicating violation of the assumption of normality and therefore non-parametric statistical tests were used.

The mean score for work satisfaction was 7.802 (SD 1.96) and the mean score for intention to stay was 8.097 (SD 2.59). Work satisfaction, intention to stay and the psychological safety questions were single or two-item measures, therefore tool reliability could not be tested.

Measures of reliability (Cronbach’s alpha (α)) were acceptable (α > 0.7) [72] for the remainder of the survey tools. WHO-5 α = 0.874; ACTA α = 0.798; UWES-3 α = 0.798; MBI α = 0.715.

#### 3.2.2. Examination of Study Concepts

##### Work Satisfaction and Intention to Stay

Single items scored out of 10 for both work satisfaction and intention to stay, were analysed as follows: scores of 9 and 10 indicated very satisfied/high intention to stay; scores of 7 and 8 indicated neutral satisfaction or undecided intention to stay and scores 1 to 6 indicated unsatisfied/intention to leave [78]. Most nurses were very satisfied (*n* = 216, 40.3%) or neutral (*n* = 229, 42.7%), whilst 17% (*n* = 91) reported low work satisfaction (M = 7.80, SD = 1.96). Similarly, most nurses reported high intention to stay (*n* = 334, 62.3%), although 14.7% (*n* = 79) were undecided, and 22.9% (*n* = 123) indicated an intention to leave their role (M = 8.10, SD = 2.60).

##### WHO Well-Being Index

Almost a third, (32.2% *n* = 174) of nurses reported well-being scores ≤52%, indicative of low well-being and possible depression [70].

##### Autonomy and Competence in Technology Adoption

Similar numbers of nurses scored a negative RAI (*n* = 248, 45.9%) compared to a positive RAI (*n* = 250, 46.3%), with 7.8% (*n* = 42) reporting a RAI score of zero, indicating equal intrinsic and extrinsic motivation. The mean perceived competence (PC) score calculated for this sample was relatively high at 3.35 out of 5 (SD = 1.07) [10].

##### Utrecht Work Engagement Scale

Mean scores (out of 6) were lowest for the vigour dimension (M = 3.39, SD = 1.06), compared to 4.29 for the dedication dimension (SD = 1.09) and 4.23 for the absorption dimension (SD = 1.09) [69].

##### Maslach Burnout Inventory

Measures using the MBI indicated 28.7% (*n* = 155) of nurses were experiencing burnout and 34.3% (*n* = 185) were engaged in their work [71].

##### Psychological Safety

Psychological safety and career trajectories satisfaction were moderate with mean scores of 2.91 (SD = 0.772) and 3.65 (SD = 0.833) (out of 5) respectively [68].

#### 3.2.3. Relationships between Study Variables

Relationships between variables were explored using Spearman’s rho (Table 2).

Whilst many relationships were statistically significant, most notable were the positive relationships between: work satisfaction and intention to stay, work satisfaction and well-being, work satisfaction and engagement, well-being and RAI, well-being and engagement, and burnout and age.

There were also several significant negative relationships measured between: work satisfaction and burnout, intention to stay and burnout, well-being and burnout, RAI and burnout, and engagement and burnout.

#### 3.2.4. Relationships between Study Variables and Nurse Characteristics

Relationships between nurse characteristics (age brackets, gender, nurse classification, years worked as a nurse, highest level of education, hours worked, work location and site of the healthcare organisation) and the study variables were tested using Pearson Chi-Square Test for Independence. Table 3 presents the results of these tests. Several relationships between groups of nurses were statistically significant. Of note were the statistically significant relationships between: RAI and age, RAI and nurse classification, and RAI and highest level of education. In this sample of nurses, both engagement and burnout also had statistically significant relationships with highest level of education, and more hours worked was statistically significant for burnout.

### 3.3. Focus Group Interviews

Data were coded and grouped into six subthemes informing two main themes: (1) Us and Them and (2) Stuck in the Middle. Table 4 presents the two themes and six subthemes. The focus group number (FG) and participant (P) reference are provided for each quote.

#### 3.3.1. Theme 1: Us and Them

The first theme, *Us and Them*, describes the feelings of ”Us” expressed by the nurses as togetherness as a workforce, when facing ”Them” as the collective for those implementing the EMR system at their healthcare organisation. This theme reflects the juxtaposition that nurses felt between their professional self-determination, and the loss of power in EMR implementation, perceived as being imposed on them and their work. Three subthemes elaborated these ideas.

##### Subtheme 1: Support, Support, Support

This subtheme captured nurses’ perceptions of support during the EMR implementation; EMR as supporting their nursing work and being supported in their work to use the EMR. 

Nurses reflected on the opportunity for the EMR to support them in their work and identified a wide range of current work practices, gaps in current practices, and frustrations they anticipated would benefit from the EMR system implementation. This included reducing the missing information experienced with paper-based medical record: “Or you miss things…’cause they get like they fall through the cracks” (FG2 P2). Nurses also anticipated the EMR would support them with patient safety concerns such as reducing medication error attributed to poor handwriting and transcription errors: “It’s a huge thing. I was reading a drug chart yesterday and there was one drug written up and I couldn’t read what it was” (FG4 P1). Communication errors or miscommunication of information was similarly expected to improve with the EMR implementation: “With everybody being on the same page, in regards to consistent communication, rather than having one team saying this, but then not being communicated to the primary treating team” (FG4 P1). It was also anticipated that EMR would save nursing time spent looking for information: “We have to chase after a certain medication, chase after certain information and that back and forth communication takes quite a lot of time sometimes” (FG2 P2).

Nurses also discussed their need for support to use the EMR, both during and after the implementation period. Nurses’ support needs often focused on education and training: “And I guess a good, you know, education for it. At least like the good introductory. I know, we have the stuff that’s online, and you can only do so much online unless you have it in front of you while you’re doing it” (FG5 P3); “I think different people learn different ways… I think that’s nice we have enough training and give us some time to settle in and get used to it I think” (FG2 P2). Nurses offered differing opinions about the training and education offered by the healthcare organisation: “Oh we’re all just I think overwhelmed, because EMR’s, the one-day training was all crammed into one day. I felt like I needed to be like, six days… wasn’t dedicated to specific wards” (FG12 P1). Nurses also identified different preferences for learning styles and information delivery that needed to be considered for education and training.

Nurses also identified nurse ”super users” as a supportive resource. These nurses had undergone advanced EMR training and were supernumerary to support their colleagues: “I think it’s good that they’re gonna have like super users that come around and actually help the nursing staff“ (FG4 P1). Mixed expectations of super users when supporting nurses were expressed: “It is nice that they’re putting on the super users or champions… on the extra shifts to be around to support. But are they trained enough?... they’re our own staff too, which is nice that it’s people we’re familiar with, you won’t feel silly asking them but will they know enough help everyone?” (FG12 P2). 

Nurses stated that they provided support and reassurance to one another when discussing their experiences and concerns about the EMR, suggesting peers were an important source of support: “[I’ve] been happy with the involvement, the education what’s available on the intranet, that any staff member at any day can just look on the intranet and hopefully get some up-to-date information” (FG2 P1); “there’s already some training available but the face-to-face, they have to prioritise the [Site F] people first because they’re going live” (FG1 P2).

##### Subtheme 2: Nurses’ Concerns

This subtheme captured nurses’ concerns about the potential for negative effects of EMR implementation on their colleagues, and the broader workforce. Nurses expressed concerns for specific groups of their colleagues, how they may feel, cope, adapt or react to the EMR implementation. Stereotyping of nurse groups was often evident. For example, a common perception that emerged was that young or junior nursing groups can quickly and easily adapt to new technology, such as the EMR, because of their newness to the nursing profession, high exposure to technology, and previous experience with EMR from placements as nursing students: “It’ll be really good for the new grads that are coming through…now they do like a week of placement and a week of uni [university] so they’re gonna just come in and smash EMR as a grad [new graduate]” (FG9 P2). 

Alternatively, older nurses were frequently identified as a group expected to experience difficulty with using the EMR system. Contributing factors included their perceived lack of general technology experience, and reduced ability to adapt to workplace changes: “I noticed that in my EMR group as well that they [older nurses] were really struggling, that they’re feeling overwhelmed by it” (FG7 P3); “But it is harder for us to pick up? Of course, it is, you know at our age we didn’t grow up like you’ve grown up with computers from the time you’ve been in school, we didn’t so obviously, it is going to be a more of a challenge for us, but I mean, you know, we work here and we’ll be supported” (FG1 P8). Some nurses voiced concerns about older nurses retiring or leaving the workplace, taking with them valuable years of work experience, due to challenges of using the EMR: “I give them credit, a lot of them are saying we’ll stay and we’ll give it a go, but if it doesn’t fall into place for them, it’s sort of the last nail sort of thing, for them” (FG7 P3); “I’m sad for the nurses that have 45 years of knowledge, and if this is going to be a barrier for them to come into work, I’m worried that we’re going to lose a lot of our senior staff” (FG7 P4). However, not all older nurses who participated in the focus groups agreed with these views, with one older nurse noting, “We actually could be computer wizzes, couldn’t we” (FG1 P8).

In addition to concern for specific groups of nurses, participants raised concerns for the nursing workforce in general. They made links between the EMR and intention of staff to stay in nursing roles, which reflected the perceived risk of nurses leaving the workforce due to the EMR implementation: “I wouldn’t be surprised if there were people resigned because it’s too hard for them. I really wouldn’t be surprised. … it feels unmanageable for some people to adopt this kind of technology, it’s a big deal, and that’s a real shame, … just because a person’s not computer literate doesn’t mean they’re not an enormous wealth of, you know, value to our organisation you know, in whatever role what they do.” (FG4 P2). Some participants suggested nurses were not adaptable: “But again there is (sic) people that are change resistors, that might take a bit longer” (FG5 P5). Participants discussed the need for resilience and ensuring the well-being and support for themselves and other nurses, especially for those who were going to be super users: “I think we all help each other out where we can, so people will sort of, we will show each other things that we know” (FG12 P2); “[It] will be quite exhausting for certain people being super users” (FG10 P1). Nurses reflected on different attitudes and ability to cope with changes in the workplace: “A lot of nurses are very steady, don’t really like change and are quite resistant to change… embracing the change and all that goes along with it will be quite difficult for some people, some personalities you know” (FG5 P5); “Well, I hope so, I think it helps. I mean we pick up on these kind of things pretty quick anyway so” (FG5 P2). Participants often cited previous experiences of new technology adoption experienced within the healthcare organization: “We all know that we’re all in it together well, we’re just going to be a bit lost but like it’s just the way individually people handle it and it’s being also respectful of that ‘cause there’s some people that it’s just gonna be the end of the world for” (FG9 P4). 

##### Subtheme 3: Ready or Not, Here Comes the EMR

The final subtheme in Us and Them was *Ready or not, here comes the EMR*. This subtheme captured nurses’ mixed feelings about their readiness for the EMR. Nurses discussed experiences of previous workplace changes as comparators for the EMR implementation. They reflected perceptions the healthcare organisation needed to keep up-to-date with other organisations; and EMRs were widespread and inevitable. Many found the lead up to the EMR implementation was a stressful experience, but anticipated that once the EMR was implemented their anxiety and stress levels would decrease.

Nurses frequently compared the EMR to previous experiences of technology related workplace changes, for example: “We all thought smart pumps were scary until they came in … and now we don’t think twice about it” (FG11 P2). These experiences were often used as reminders to other nurses in the group about their success, and to reassure their colleagues. 

Nurses also discussed how the EMR implementation was not optional; rather, it was perceived as an organisational change they had to go through. They acknowledged it was necessary and would help the organisation and their profession to be up-to-date with contemporary advancements in technology, locally and internationally: “You know it’s the way every hospital is going so it’s, it was only a matter of time before we probably did” (FG7 P6); “Keep up with the world” (FG11 P4). 

Nurses expressed emotions that ranged from excited, anxious and uncertain, and dread: “I’m excited, I think it will be great” (FG7 P2); “Not excited, just 90% anxious“ (FG9 P6); “I don’t think you’ll ever be ready, you’re just gotta get, jump into the deep” (FG8 P1). Many were unsure about how the EMR would be operationalised in their clinical setting, and how they could prepare themselves. Nurses who were uncertain about their readiness for EMR often indicated they had no prior EMR experience: “I wouldn’t have a clue, I’ve never used an EMR before” (FG11 P1). Alternatively, those with previous EMR experience more often expressed excitement and enthusiasm: “Like when I’ve used it in the past, it was, I didn’t really have any complaints so yeah” (FG6 P4). Such emotional responses appeared to reflect varying levels of self-readiness that were the accumulation of engagement, training, education and discussions about the soon-to-be-implemented EMR system across the organisation. Nurses reporting little previous EMR experience indicated they found the preparation for the EMR more stressful than they expected the reality of EMR to be: “Yeah I’m excited too I just feel like the build up’s been worse than the actual roll out” (FG8 P1). Many nurses wanted the EMR implemented so the stressors of implementation would be over. Alternatively, nurses working at the sites with implementation scheduled later were glad to be in this position, reassured the EMR would be functioning well when they received it: “I’m sure once it gets going and the teething problems, by the time it gets to us, hopefully, they’re sorted” (FG3 P4).

#### 3.3.2. Theme 2: Stuck in the Middle with EMR

The second theme was titled *Stuck in the middle with EMR*. This theme represents nurses’ feelings and expectations about changes the EMR system would have on the patient-nurse dyad. Nurses wondered whether they would need to choose between attending to the EMR and their patients. They also identified the potential for the EMR to create competing priorities in the clinical setting that may impact their nursing work and ability to provide safe and quality patient care. The three subthemes are described below.

##### Subtheme 1: Ultimately, Patient Care Is the Most Important Thing

This subtheme captured nurses’ perceptions of what was most important, or what mattered most to them in regard to the EMR implementation. Nurses stated their ability to provide safe and quality patient care was the most important thing to them: “Ultimately, that’s the most important thing yeah”, referring to patient safety (FG7 P3). They viewed the EMR as a supportive resource that they could leverage to improve patient care: “Hopefully improve patient-centered care” (FG3 P2); “I think on how it will improve both nursing practice or medical practice, and also, most importantly, patient safety” (FG6 P3).

Through this discussion, the safety of patients during the EMR implementation was voiced as an important nursing responsibility. Nurses anticipated a need to balance patients’ needs with the demands of the computer: “I think it will make you more vigilant because you’re like come on where am I looking” (FG11 S9); “And try not to get so caught up on it without jeopardising that patient care” (FG11 S7). Managing patient experience and the perception of patients and their families about EMR were also identified as important nursing considerations: “But the patient experience I think will actually improve when we’re all communicating in a real-time fashion and…not … having to ask multiple questions” (FG2 P1); “I would hate for parents to be worried about their baby not being cared for, because everyone’s like oh my God it takes so long to put something in, I would hate for a parent to think oh they’re so busy on the computer, who’s actually watching my baby” (FG7 P3).

##### Subtheme 2: Great Expectations

*Great expectations* reflects nurses’ expectations about how the EMR will change nursing work and workflows. Expected benefits of the EMR included improved medication safety, less duplication of information, legibility of the patient medical record, ability to provide clinical support, decreased use of paper, and improved access to patient information: “But also might help you to make decisions about a certain thing or prompt you to … recognise when your obs [patient observations] are outside of their parameters … have a different way of alerting you” (FG4 P2); “Less duplication, definitely” (FG1 P6); “I think it’ll be good for legibility of writing … we aren’t always clear and as legible for other people to interpret what we’ve written“(FG2 P1); “Real time information that’s going in as it’s happening” (FG7 P4); “So from an efficiency point of view,… multiple people can access a medical record at one time” (FG4 P2); “Maybe like less medication errors and stuff as well, because, … you’re getting prompted and scanning their barcodes” (FG8 P3); “I can see the weight, and the patients’ blood results, whether it’s actually the right dose…that sort of thing. So it’s safer for the patient” (FG1 P2); “Also, environmentally friendly. ‘Cause we’re not using as much paper now” (FG9 P5). 

Some nurses were not enthusiastic about ideas such minimising paper used: “But we will need a drawer for paperwork” (FG11 P1); “We definitely will still need a bed folder” (FG11 P2).

Some nurses discussed expectations about a future EMR system that included retaining some paper (such as consent forms): “… theatre aren’t on EMR as well. So we’re still going to have some paper trail, and then some electronic so it’s ensuring that is aligned as well and there’s no double up” (FG3 P4).

Nurses made recommendations for implementation. Future ideals discussed included device integration, ways the system could be used to benefit nurses and patients, and suggestions to improve data security: “How about that EMR app will only work within the facility on the WiFi but not outside so you can’t breach. That’d be an ideal” (FG10 P1); “The transfer over of notes from hospitals, like different hospitals, it should just all be one big database. GPs, everything” (FG6 P1); “Hopefully in the future, …there might be enough money … that our machines could talk directly to EMR” (FG2 P1).

##### Subtheme 3: Weight of the (EMR) World

*Weight of the (EMR) world* reflected nurses’ feelings of concern, worry about aspects of EMR implementation; for example, what happens if the EMR isn’t working, the amount and type of hardware to be provided, the usability of the system, and a mismatch between nurses’ clinical work and the EMR system would interfere with their nursing work. Nurses were often cynical about changes, expressed in negative comments about organisational and individual responses to change, data security, and oversight of the system.

Nurses frequently discussed the number and type of devices to be delivered to the clinical areas, whether the software would be fast enough to do their work or slow computers would impact on their time with their patients: “I guess our, one of our fears here is that we won’t have enough pieces of equipment” (FG3 P4); “I suppose just technology actually working and doing what it’s meant to do … timing … to log on and log off and freezing and not freezing” (FG11 P1). Nurses also expressed concern about what would happen if the system went down, if the layout, design and language of the software were difficult because of its American origins: “Maybe like a good backup plan for when it does crash or like when something bad goes wrong” (FG8 P3); “And then go into EMR it’s all different wording, it’s all American, it’s not actually Australian phrases, or English phrases” (FG12 P1).

Nurses were deeply concerned about potential for negative impacts of the EMR on their nursing work and workflows: “That’s my worry, … you’ve got…all this stuff and you have to log onto the computer to figure it out, …, find the orders and you can’t just do it, I’m worried that the baby will be compromised because of the computer” (FG7 P4); “Although, I feel like we’re sort of giving half of our nursing brain away … like you learnt so much … and now the machine’s just gonna do it for you” (FG6 P1). At times, the computer was identified as a separate entity or intruder into the familiar nurse-patient dyad for nurses to navigate or negotiate when they provide patient care. Oversight of the system and data security were frequently raised as making nurses nervous about the implications for their practice: “Big Brother will know who makes mistakes, and who doesn’t make mistakes” (FG12 P1); “But at the end, it has to be documented, because if we get called into the coroners, you know, it is what it is” (FG7 P4); “I’m sure they have a good like security thing but they haven’t told us … it would be good to know ‘cause …. you could be exposed to whatever” (FG10 P1).

## 4. Discussion

Independent analyses of surveys and focus group data provided insights into the complex concepts of nurses’ motivation, engagement and well-being in the context of preparing for a significant workplace change of EMR implementation. Generally, nurses were positive about adoption of the EMR in their workplace and indicated they expected the EMR would facilitate their work in meaningful ways, such as timely and legible documentation, and provide support to improve patient safety and care delivery. These expectations were consistent with previous literature published on nurses’ expectations of EMR systems [16,79,80].

Although many nurses indicated they were engaged and satisfied in their work, evidence of low well-being and burnout, combined with comments about stress, anxiety and uncertainty, suggests some nurses may be vulnerable to negative impacts of the EMR implementation.

### 4.1. Nurses Were Engaged and Satisfied in Their Work

Measurement of work engagement in three ways revealed consistent findings, enhancing their validity. The single-item global measure and work satisfaction scores from the UWES-3 similarly showed good work satisfaction, and adequate mean scores in the dimension of vigour, and good levels for dedication and absorption. Fewer nurses were classified as engaged using the MBI tool’s indicator for work engagement, which corresponds to literature on interpretations of work engagement using the MBI and UWES-3 tools [27]. These survey results were consistent with Theme 2 (Stuck in the Middle with EMR) from the qualitative data, where nurses indicated their work engagement and work satisfaction were underpinned by their desire to provide high-quality patient care.

Whilst the reported levels of psychological safety and career trajectories corresponded to the survey participants’ work satisfaction results and high intention to stay scores, the overall findings indicated some nurses were experiencing challenges in their work. The voicing of current work challenges, as well as the implications the EMR implementation may had for patient safety, reflected the strong desire for nurses to provide the best care possible for their patients, and appeared to underpin their anxiety and vulnerability about the potential impact an EMR system would have on their patient-nurse relationship.

### 4.2. Some Nurses May Be Vulnerable to the Negative Impacts of EMR Implementation

The potential for vulnerability of some nurses to the negative impacts of the EMR was made visible in the survey results and in Theme 1 (Us and Them). Nurses expressed their concerns about their low sense of control over the EMR implementation, despite good organisational resources and engagement. Nurses’ competence, or feeling capable in one’s work, role or actions were measured by the ACTA tool (PC subscale) indicating nurses believed they would be able to use the EMR [10]. This score was found to be relatively high in this sample, and statistically significant positive correlations between RAI and work satisfaction, intention to stay, work engagement and well-being indicated that as nurses perceived a sense of control over the EMR, they were more satisfied, engaged and likely to stay in their roles, which corresponds to previous literature [81]. As expected, well-being had a statistically significant positive correlation with work engagement and statistically significant negative correlation with burnout. Whilst in turn, burnout was found have a statistically significant negative correlation with well-being. This corresponds to previous literature published on intensive care unit nurses’ perceptions of an EMR which found EMR satisfaction was linked to the nurses’ well-being [82].

Similarly, statistically significant negative correlations between RAI and burnout, nurses’ age and years worked, suggested that as RAI decreased, the other measures increased. This negative correlation between RAI and burnout differs to reports in the literature which suggest a perception of higher control (more positive RAI) was associated with higher burnout [81]. Interestingly, the RAI also had a statistically significant association with nurses’ age, classification and years worked, albeit with small effect sizes.

In this sample, younger nurses worked significantly more hours and had been working for less time compared to their older nurse counterparts. Alternatively, older nurses had worked for longer, worked less hours, and had more burnout. A study on intensive care unit nurses’ satisfaction of an EMR system found that older nurses were more dissatisfied when spending more time on the EMR than their younger colleagues [82]. Though the transferability of the study findings may not be relevant to an organisation-wide nursing population, the potential negative impacts of the EMR implementation on specific groups such as older nurses should be noted, and were voiced by nurses in this sample. Concerns regarding the possibility of older colleagues leaving the profession due to stressors of the EMR implementation related to lack of familiarity with technology or adapting to change, and nurses were concerned about their colleagues’ well-being and the potential knowledge deficit that would result if these experienced and valued nurses left the workplace. A recent scoping review revealed that older nurses face specific challenges at work such as fatigue, not feeling valued at work, and being treated differently to their younger colleagues [83]. In this sample older nurses were regarded as highly respected and valued by their colleagues, however older nurses themselves did not always feel as such. This further supports the need for the older nursing workforce to be supported and feel valued during a period of transformative change such as the EMR implementation.

Focus group participants voiced concern about the current state of nurses’ well-being with the anticipation of the EMR implementation, and the potential for any further negative impacts on nurses. Nurses stated they were anxious and stressed, which corresponded with survey results signifying just over a third of the nurses had scores indicative of low well-being and possible depression, and just under a third of nurses were found to be experiencing burnout. Well-being has been found to be predictive of nursing turnover [84], and previous studies report work engagement, work satisfaction and intention to stay are inter-related, with low work satisfaction associated with low intention to stay and low work engagement, and vice versa [19,85]. Similarly, in this sample, the statistically significant positive correlations between intention to stay and well-being and engagement, and a statistically significant negative correlation between intention to stay and burnout indicate that nurses were less likely to leave the profession if they were engaged in their work and not experiencing burnout. 

Work satisfaction and efficiency can also be measured by examining perceptions of psychological safety [68]. This sample of nurses demonstrated comradery and collegial support in the focus group interview data by expressing concerns over their colleagues. However, there were also mentions of fear of negative consequences and cynicism when discussing oversight of the system, needing help from others, or making mistakes as a new user of the EMR. Work satisfaction had statistically significant positive correlations with intention to stay, well-being and engagement, whilst it had statistically significant negative correlations with burnout, nurses’ age and years worked. Survey data on work satisfaction in this population corresponded to the focus group interviews and previous literature in which nurses reported concerns about work stressors such as PC with the EMR system or older nurses leaving the profession [86].

Nurses also identified strategies and needs they believed may help alleviate the negative impacts of the EMR implementation. The overarching concerns regarding vulnerable nurses included the potential for the EMR not to be used properly or at all, the potential to lose nurses from the workforce and the view that nurses may not feel they are able to do their jobs. These concerns correspond with existing literature in which authors assert the importance of nurses’ feeling engaged in their work, the links between nurse engagement and patient safety outcomes, as well as the need to attempt to mitigate the potential negative consequences of an EMR on the nursing workforce [4,13,23].

### 4.3. Implications for Practice

Understanding nurses’ key concerns can assist EMR implementation and ongoing optimisation strategies in ways that mitigate potential for negative consequences for nurses, patient safety and organisational outcomes. Some nursing groups may be vulnerable to negative impacts or increases in stress associated with an EMR implementation. Nurses with concerns about their competence with the EMR system, or who have negative attitudes towards the EMR, may benefit from tailored education or support such as protected time with colleagues to review unfamiliar aspects of the EMR system. Suggestions include obtaining specific feedback from nursing staff about their work, such as whether the EMR is impacting their patient care delivery, asking them about ideas for improvements, and acting to ensure supportive and accessible measures are in place to prevent nurses leaving the workforce due to EMR-related concerns.

### 4.4. Limitations and Reflexivity

The relatively low survey response rate is a limitation of this study. Multiple strategies advocated by experts had limited success with this population. It is possible the different response options (online, QR code or on paper), may have impacted responses or data quality, but minimal missing data suggests this is not the case. 

This study was undertaken concurrently with the natural experiment of an organisation-wide EMR implementation and therefore it was not possible to use an experimental study design or control groups.

At the commencement of this study, the timing for implementation of the EMR had been delayed, which may have also led to staff frustration contributing to a response bias. The timing of data collection across multiple clinical areas and multiple sites also meant that some nurses had completed the survey and/or participated in the focus groups after they had completed EMR training, and some had not. The inclusion of nurses who had undergone the training as well as those who had not, may be considered a strength of the study as it ensured diverse participant views were captured. 

The convenience sampling used for recruitment of focus group interview participants was another potential source for response bias, but similarities between survey and focus group participants suggests this risk was minimal. Convenience sampling was ideal to manage complex issues of recruiting participants working in a large healthcare organisation. All the focus group interviews were held during the staff overlap between morning and afternoon shifts as this facilitated nurses attending during work time. However, competing clinical and organisational priorities often meant that as it got close to the EMR implementation time it became more difficult to find available rooms, and have nurses released from other duties to attend focus group interviews at the allocated times. In addition, participant availability at different times and sites, and their desire versus the convenience to participate were other recruitment considerations. In addition, flexibility in data collection timing and open communication were critical to mitigate recruitment and data collection challenges.

The mix of staff seniority was another factor that may have impacted data quality. The contributions of junior nurses did not appear to be impacted by the presence of senior staff, such as associate nurse managers or nurse managers, in focus groups. Conversely, leaders appeared to raise area-specific issues or concerns, such as hardware or training as well as voicing concerns or apprehension about the EMR implementation. Most of the focus groups included diverse participants that added to data richness. 

## 5. Conclusions

Despite satisfaction in their work, the addition of a new healthcare technology in the form of an EMR system is another stressor for nursing staff, even when it has not yet been implemented. EMR implementations must therefore be carefully managed and monitored in nurses’ complex work environments, to ameliorate potential for direct impacts on nursing care delivery and patient safety. The findings of this study support the need to involve stakeholders at all stages of implementing new technologies into healthcare environments. There is a need for continuous evaluation to respond to expected and unexpected consequences. Further research examining the post-implementation effects of the EMR must be undertaken to fully understand the complexity of factors associated with technology implementation, and the complexity of factors that influence well-being, motivation, engagement, intention to stay and burnout in nurses. The findings of this study support the need for post-EMR implementation research to enable comparison of results.

## Figures and Tables

**Table 1:** Survey Measures, tools used, number of items and scoring.

Concept	Survey Measures	Tool and Tool Dimensions	Number of Items	Scoring
Motivation	EMR Autonomy and Competence	ACTA (Autonomy and Competence in Technology Adoption) [10] ^1^ Tool dimensions: Perceived Competence and Relative Autonomy Index (RAI)	14	5-point Likert scale (1 Not all the time–5 Very true)
Engagement	Work Satisfaction	Single-item measure [66,67]	1	Score out of 10 (1–10)
		Psychological Safety questions (adapted) [68] Tool dimensions: Team Safety, Work Satisfaction	3	5-point Likert scale (0 Strongly disagree–4 Strongly agree)
	Intention to Stay	Single-item measure [66,67] ^2^	1	Score out of 10 (1–10)
	Work Engagement	UWES-3 (Utrecht Work Engagement Scale) [69] Tool dimensions: Vigour, Dedication, Absorption	3	7-point Likert scale (0 Never–6 Always)
Well-Being	Well-Being	WHO-5 (Well-Being Index) [70]	5	6-point Likert scale (0 at no time–5 All of the time)
	Burnout	MBI (Maslach Burnout Inventory) [71] ^3^ Tool dimensions: Exhaustion, Cynicism, Efficiency	9	7-point Likert scale (0 Never–6 Always)
Demographics	Age Gender Nurse classification Years worked as a nurse Highest level of education Hours worked Work location Site of the healthcare organisation	-	-	-

^1^ Permission to use ACTA given by R.C.; ^2^ Measured as intention to leave (single-item measure) then reverse-scored for analysis. ^3^ Permission to use MBI given by M.L.

**Table 2:** Descriptive Statistics and Correlation Matrix–Spearman’s rho (N = 468).

	Mean	Standard Deviation	Work Satisfaction	Intention to Stay	WHO-5 Cutoff	ACTA Relative Autonomy Index	MBI–Engagement	MBI–Burnout	Age	Years Worked as a Nurse	Hours Worked
Work Satisfaction	7.895	1.908	1.000	-	-	-	-	-	-	-	-
Intention to Stay	8.170	2.554	0.382 **	1.000	-	-	-	-	-	-	-
Well-Being	1.690	0.463	0.380 **	0.200 **	1.000	-	-	-	-	-	-
Relative Autonomy Index	1.508	0.500	0.176 **	0.105 *	0.192 **	1.000	-	-	-	-	-
MBI–Engagement	0.331	0.471	0.124 **	0.120 **	0.108 *	0.129 **	1.000	-	-	-	-
Burnout	0.284	0.452	−0.183 **	−0.192 **	−0.234 **	−0.110 *	−0.443 **	1.000	-	-	-
Age	37.68	11.760	−0.104 *	−0.025	−0.047	−0.127 **	−0.035	0.091 *	1.000	-	-
Years worked as a nurse	13.55	11.528	−0.147 **	−0.027	−0.053	−0.187 **	−0.084	0.142 **	0.870 **	1.000	-
Hours worked	58.89	18.111	0.051	0.018	0.014	0.006	−0.043	0.086	−0.099 *	−0.122 **	1.000

** Correlation is significant at the 0.01 level (2-tailed). * Correlation is significant at the 0.05 level (2-tailed). Bootstrap results based on 1000 bootstrap samples. WHO-5 = Well-Being Index. ACTA = Autonomy and Competence in Technology Adoption. MBI = Maslach Burnout Inventory.

**Table 3:** Group variables–Pearson Chi-Square Test for Independence (N = 466).

	Age	Nurse Classification	Highest Level of Education	Hours Worked
Work Satisfaction	χ^2^ = 14.714, df = 8 *p* = 0.065 Cramer’s V = 0.126	-	-	-
Intention to Stay	χ^2^ = 1.622, df = 8 *p* = 0.990 Cramer’s V = 0.042	-	-	-
Well-Being	χ^2^ = 5.768, df = 4	-	χ^2^ = 4.223, df = 4	χ^2^ = 6.095, df = 5
	*p* = 0.217		*p* = 0.377	*p* = 0.297
	Cramer’s V = 0.111		Cramer’s V = 0.095	Cramer’s V = 0.114
Relative Autonomy Index	χ^2^ =15.381, df = 4	χ^2^ =18.664, df = 7	χ^2^ =15.179, d	χ^2^ = 3.185, df = 5
	*p* = 0.004 *	*p* = 0.009 *	*p* = 0.004 *	*p* = 0.672
	Cramer’s V = 0.182	Cramer’s V = 0.200	Cramer’s V = 0.180	Cramer’s V = 0.083
Engagement	χ^2^ =3.181, df = 4	-	χ^2^ = 20.144, df = 4	χ^2^ = 4.569, df = 5
	*p* = 0.528		*p* < 0.001 *	*p* = 0.471
	Cramer’s V = 0.083		Cramer’s V = 0.208	Cramer’s V = 0.099
Burnout	χ^2^ = 7.116, df = 4	-	χ^2^ = 3 3.669, df = 4	χ^2^ = 14.000, df = 5
	*p* = 0.130		*p* ≤ 0.001 *	*p* = 0.016 *
	Cramer’s V = 0.124		Cramer’s V = 0.269	Cramer’s V = 0.173

Cells that do not include data in this table are those that violated assumptions and are therefore not included as they cannot be accurately interpreted. * *p*-value ≤0.05. χ^2^ = Pearson chi-square. df = degrees of freedom. Cramer’s V = effect size (0.1–0.29 = small).

**Table 4:** Themes and Subthemes.

Theme	Subthemes
1. Us and Them	1. Support, support, support
	2. Nurses’ concerns
	3. Ready or not, here comes the EMR
2. Stuck in the middle with EMR	1. Ultimately, patient care is the most important thing
	2. Great expectations
	3. Weight of the (EMR) world

## Data Availability

The data presented in this study are available from the corresponding author upon reasonable request. The data are not publicly available due to privacy and ethical restrictions.

## Supplementary Materials

The following are available online at https://www.mdpi.com/1660-4601/18/5/2726/s1, Table S1: Demographic Characteristics of Survey and Focus Group Participants.
