# Supplementary material for: Nurse Motivation, Engagement and Well-Being before an Electronic Medical Record System Implementation: A Mixed Methods Study
Source: Int J Environ Res Public Health. 2021 Mar 8;18(5):2726. doi: 10.3390/ijerph18052726 (PMC7967448; doi:10.3390/ijerph18052726)
Supplement: Supplementary file 1 [file ijerph-18-02726-s001.pdf]

International Journal of
*Environmental Research
and Public Health*

MDPI

Appendix A

Table S1. Demographic Characteristics of Survey and Focus Group Participants.

Variable	Mean (SD)	Dimensions	Survey<br>Total N=540	Focus Groups<br>Total N=63
Age	37.52(11.89)	20-29	165 (30.6)	23 (36.5)
		30-39	150 (27.8)	12 (19)
		40-49	104 (19.3)	10 (15.9)
		50-59	74 (13.7)	4 (6.3)
		60-69	33 (6.1)	2 (3.2)
		Missing	14 (2.6)	12 (19)
Gender		Male	47 (8.7)	4 (6.3)
		Female	483 (89.4)	54 (85.7)
		Other/Prefer not to say	8 (1.5)	-
		Missing	2 (0.4)	5 (7.9)
Nurse classification		RN (Graduate)	56 (10.4)	9 (14.3)
		RN (Grade 2)	232 (43)	28 (44.4)
		EN	38 (7)	7 (11.1)
		CNS	89 (16.5)	1 (1.6)
		ANUM	79 (14.6)	7 (11.1)
		NM	22 (4.1)	4 (6.3)
		Educator	13 (2.4)	-
		Nurse Consultant / Nurse Practitioner	6 (1.1)	2 (3.2)
		Missing	5 (0.9)	5 (7.9)
Years worked as a nurse		0-4	128 (23.7)	23 (36.5)
		4.5-9	115 (21.3)	11 (17.5)
		10-14	83 (15.4)	5 (7.9)
		15-19	60 (11.1)	6 (9.5)
		20-24	45 (8.3)	4 (6.3)
		25-29	27 (5)	2 (3.2)
		30-34	28 (5.2)	4 (6.3)
		35-39	19 (3.5)	-
		40-44	11 (2)	1 (1.6)
		45-49	10 (1.9)	-
		50-54	2 (0.4)	-
		Missing	12 (2.2)	7 (11.1)
Highest level of Education		High School	11 (2.0)	1 (1.6)
		Certificate or Diploma	49 (9.1)	5 (7.9)
		Degree	229 (42.4)	32 (50.8)
		Postgraduate Certificate or Diploma	185 (34.3)	17 (27)
		Higher Degree (Master's or PhD)	61 (11.3)	2 (3.2)
		Missing	5 (0.9)	6 (9.5)
Hours worked (average per fortnight)	59.39(17.75)	0-16	12 (2.2)	-
		17-32	60 (11.1)	1 (1.6)
		33-48	93 (17.2)	2 (3.2)
		49-64	207 (38.3)	31 (49.2)
		65-80	136 (25.2)	20 (31.7)
		>80	17 (3.1)	1 (1.6)

	Missing	15 (2.8)	8 (12.7)
Work location	Medical / Surgical Ward	154 (28.5)	4 (6.3)
	Critical Care	222 (41.1)	15 (23.8)
	Paediatrics	77 (14.3)	11 (17.5)
	Sub-Acute	60 (11.1)	14 (22.2)
	Nurse Consultant / Nurse Practitioner	2 (0.4)	2 (3.2)
	Procedural Units	23 (4.3)	11 (17.5)
	Missing	2 (0.4)	6 (9.5)
Site	A	89 (16.5)	1 (1.6)
	B	46 (8.5)	14 (22.2)
	C	102 (18.9)	26 (41.3)
	D	141 (26.1)	6 (9.5)
	E	53 (9.8)	10 (15.9)
	F	105 (19.4)	-
	Other (not specified)	1 (0.2)	-
	Missing	3 (0.6)	6 (9.5)

SD=Standard Deviation. RN = Registered Nurse. EN = Enrolled Nurse. CNS = Clinical Nurse Specialist. ANUM = Associate Nurse Unit Manager. NM = Nurse Manager.

© 2020 by the authors. Submitted for possible open access publication under the terms and conditions of the Creative Commons Attribution (CC BY) license (http://creativecommons.org/licenses/by/4.0/).
